# The Influence of Hypoxia and pH on Bioluminescence Imaging of Luciferase-Transfected Tumor Cells and Xenografts

**DOI:** 10.1155/2013/287697

**Published:** 2013-07-07

**Authors:** Ashraf A. Khalil, Mark J. Jameson, William C. Broaddus, Peck Sun Lin, Seth M. Dever, Sarah E. Golding, Elizabeth Rosenberg, Kristoffer Valerie, Theodore D. Chung

**Affiliations:** ^1^Department of Otolaryngology-Head and Neck Surgery, University of Virginia Health System, P.O. Box 800713, Charlottesville, VA 22908-0713, USA; ^2^Department of Neurosurgery, Virginia Commonwealth University, Richmond, VA, USA; ^3^Department of Radiation Oncology, Virginia Commonwealth University, Richmond, VA, USA; ^4^Department of Radiation Oncology, Georgia Health University, Augusta, GA, USA

## Abstract

Bioluminescence imaging (BLI) is a relatively new noninvasive technology used for quantitative assessment of tumor growth and therapeutic effect in living animal models. BLI involves the generation of light by luciferase-expressing cells following administration of the substrate luciferin in the presence of oxygen and ATP. In the present study, the effects of hypoxia, hypoperfusion, and pH on BLI signal (BLS) intensity were evaluated *in vitro* using cultured cells and *in vivo* using a xenograft model in nude mice. The intensity of the BLS was significantly reduced in the presence of acute and chronic hypoxia. Changes in cell density, viability, and pH also affected BLS. Although BLI is a convenient non-invasive tool for tumor assessment, these factors should be considered when interpreting BLS intensity, especially in solid tumors that could be hypoxic due to rapid growth, inadequate blood supply, and/or treatment.

## 1. Introduction

*In vivo* bioluminescence imaging (BLI) is a technology that is frequently used in the study of animal tumor models [1]. It has been successfully used to follow many different types of tumors, such as prostate, breast, colon, ovarian, and lung cancers [2–8]. The *in vivo* BLI method is based on the action of luciferase on luciferin which produces light emission within the xenograft [9, 10]. The light-producing reaction requires molecular oxygen and ATP for the oxidation of luciferin to oxyluciferin. The light produced is transmitted through tissue and detected by a sensitive charge-coupled device (CCD) camera; the acquired data can be presented as qualitative pseudocolor images or as quantitative photon counts.

A significant advantage of *in vivo *BLI is the ability to noninvasively obtain several data points from the same group of animals by repeated monitoring. In addition, the sensitivity of *in vivo *BLI permits the detection of very small tumors or metastases [8, 11]. A major concern is that solid tumors frequently outgrow their oxygen supply and can develop central hypoxia [12]. Alternatively, tumor hypoxia can develop as a result of treatment [13]. In these settings, oxygen available for the BLI reaction could be reduced to limiting levels, which would result in a reduced BLI signal (BLS) and underestimation of the actual tumor size [14].

In the process of developing an *in vivo *BLI-based mouse model of U87 glioma cells for evaluation of radiotherapy, we noted that these solid tumors frequently become transiently or chronically hypoxic and that, in this situation, tumor growth determined by BLI may be an underestimate. We therefore present the effect of oxygen, hypoxia, pH, and cellular viability on the BLS in this model. The *in vitro* and *in vivo* results indicate that the development of hypoxia or pH changes could impact the use of BLI in quantitative studies of tumor growth and treatment response. 

## 2. Materials and Methods

### 2.1. Cell Lines

U87 human malignant glioma cells were obtained from ATCC (Manassas, VA) and were transfected with the cDNA encoding firefly luciferase to produce U87-Luc cells. These were maintained in DMEM medium (Invitrogen, Grand Island, NY) supplemented with 10% fetal bovine serum (JRH Biosciences, Lenexa, KS) and 1% penicillin/streptomycin (Invitrogen).

### 2.2. Xenograft Tumors

Xenograft tumors were generated in female nude mice 7-8 weeks old. Five million U87-Luc cells in 100 *μ*L PBS were injected subcutaneously in the dorsal side of the upper hind limb of female mice using insulin syringe. Xenografts were allowed to grow for 10 days when the initial measurement was made with calipers and with BLI. Tumor volume measurements were calculated using the formula for an oblong sphere: volume = (width^2^ × length). The mice were handled in accordance with IACUC guidelines; experiments were approved by the institutional Committee for Animal Research.

### 2.3. Bioluminescent Imaging

BLI was performed using the IVIS-200 Imaging System (Xenogen Corporation, Berkeley, CA). Mice were anesthetized by inhalation of 2% isoflurane (Abbott Laboratories, Chicago, IL). Each set of mice were positioned in the special imaging chamber and injected subcutaneously (dorsal midline) with 150 mg/kg D-luciferin (Xenogen; PerkinElmer, Waltham, MA) in approximately 200 *μ*L. The luminescent camera was set to 1 min exposure, medium binning, f/1, blocked excitation filter, and open emission filter. The photographic camera was set to 2 s exposure, medium binning, and f/8. The field of view was set at 22 cm distance to image up to 5 mice simultaneously or 4–12.9 cm to view plates and tubes. Images were acquired in sequence at 1 min intervals (60 s exposure, no time delay) for 30 min. The intensity of BLS in the luminescent area of the tumor, which is also described as the region of interest (ROI), was determined by Living Image 3D software (version 1; Xenogen). BLS was plotted as photon/sec/m^2^ against time as an indicator of tumor burden.

### 2.4. BLI after Acute Ischemia

A nude mouse with bilateral thigh U87-Luc xenografts underwent BLI as described above. While under the same general anesthesia, a rubber band was firmly tightened around the right thigh proximal to the tumor. 2 min after application of the band, BLS was collected for 1 min. The band was then removed; 2 min later, BLS was collected again for 1 min.

### 2.5. BLI after Systemic Hypoxia

A nude mouse with right thigh U87-Luc xenografts was kept in a hypoxia chamber (Bactrox, SHEL LAB, Sheldon Manufacturing Inc., Cornelius, OR) for 10 min in 95% N_2_/5% CO_2_. The mouse was injected SC with 150 mg/kg D-luciferin. General anesthesia was induced by IP injection of 10 mg/kg ketamine. The mouse was then put into a sealed transparent bag and transferred to the imaging chamber; BLI was performed as described above. Complete euthanasia was achieved by CO_2_ inhalation while still in the sealed bag. BLI was performed before and after 1 mL air was injected into the tumor using an insulin syringe.

### 2.6. *In Vitro* Hypoxia

Oxyrase (Oxyrase Inc., Mansfield, OH), which consumes oxygen directly, was used to induce acute hypoxia in the tissue culture media (final concentration 50–250 mU/mL).

## 3. Results and Discussion

**Figure 1:**
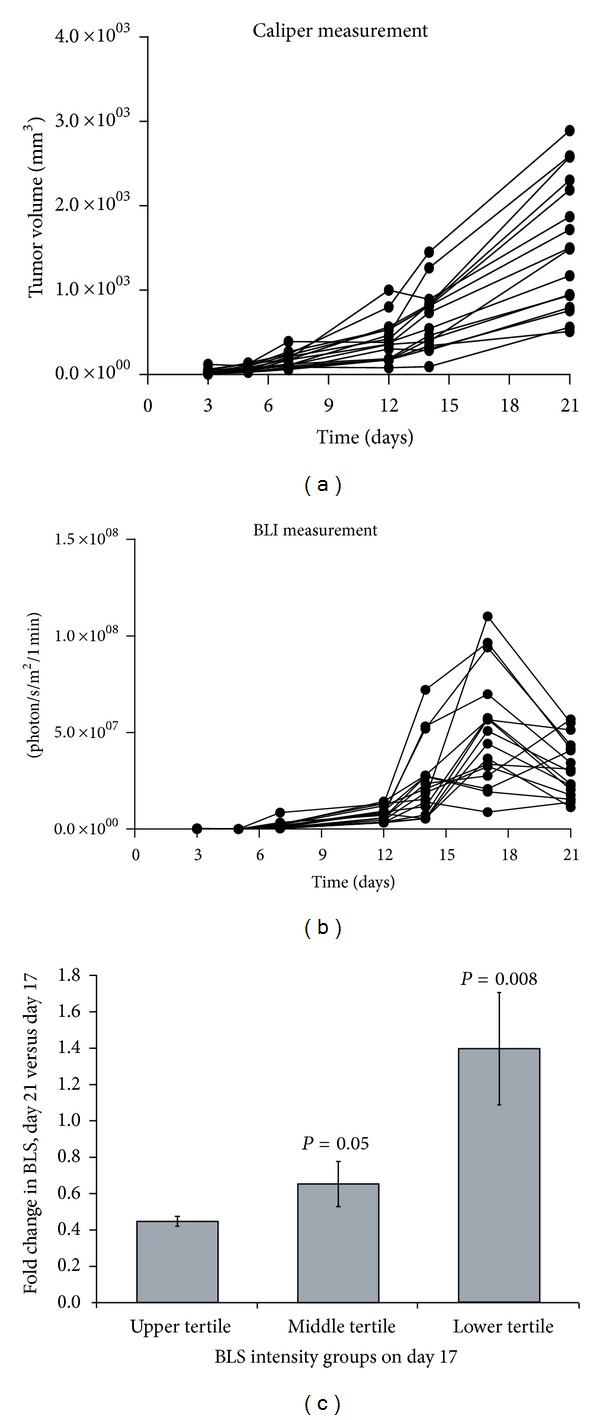
Flank xenograft growth by caliper measurement and BLI. U87-Luc flank xenografts were measured by calipers (a) and BLI (b) twice per week for 3 weeks. The tumors were allowed to achieve a large size to create central hypoxia. The tumors were segregated into upper, middle, and lower tertiles based on their BLS on day 17. For each tertile group, the average BLS on day 21 relative to day 17 was quantified as a fold change (c).

Figure 1 compares the growth of U87-Luc flank xenografts when measured by calipers (Figure 1(a)) or BLI (Figure 1(b)) and allowed to grow to a large size. The two evaluations of tumor volume were similar through day 17, but subsequently a drastic reduction in BLS was noted while volume calculated using caliper measurements continued to increase. As shown in Figure 1(c), the fold change in BLS on day 21 relative to day 17 was dependent on tumor size. Specifically, tumors that had the weakest BLS on day 17 (lower tertile) demonstrated, on average, a 38.7% increase in BLS on day 21. Conversely, tumors with the most intense BLS on day 17 (upper tertile) showed an average of 55.8% reduction in BLS on day 21. Regardless of tumor size on day 21, maximal BLS intensity always occurred at the center of the tumor. Additionally, some tumors were removed and sectioned immediately after euthanasia; these did not show any gross evidence of central necrosis. These findings suggest that the reduction in BLS on day 21 was not a result of central tumor necrosis. Thus, the correlation of change in BLS with tumor size suggests that the BLS is impacted by physiologic tumor changes that correlate with increasing size, such as hypoxia and/or pH change. In order to investigate this phenomenon, we performed a series of studies using BLI on cultured cells *in vitro*. 

**Figure 2:**
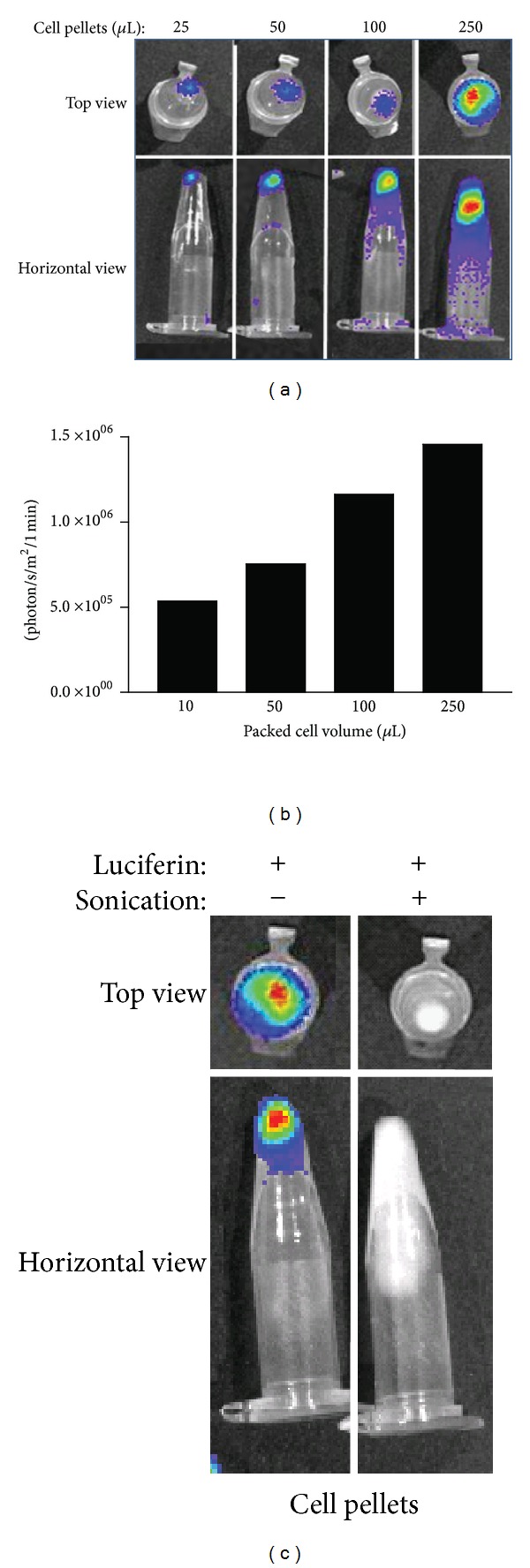
Impact of cell number and cell viability on BLS. Aliquots of U87-Luc cells (500 × 10^3^ cells/*μ*L) were pelleted in conical tubes, and BLI was performed ((a) image; (b) quantification). Two equal aliquots of U87-Luc cells were evaluated by BLI after one sample was sonicated for 1 min (c).

It is well established that BLS intensity correlates with cell number. To demonstrate this for U87-Luc cells, aliquots pelleted in conical tubes were assessed by BLI (Figure 2(a)). Intensity of the BLS correlated with total cell number (Figure 2(b)). To test the effect of viability on signal generation, equal volumes of either healthy, viable or sonicated cells were exposed to luciferin and the BLS was collected for 1 min. Sonicated cells showed no light emission indicating that only intact cells can produce BLS (Figure 2(c)).

**Figure 3:**
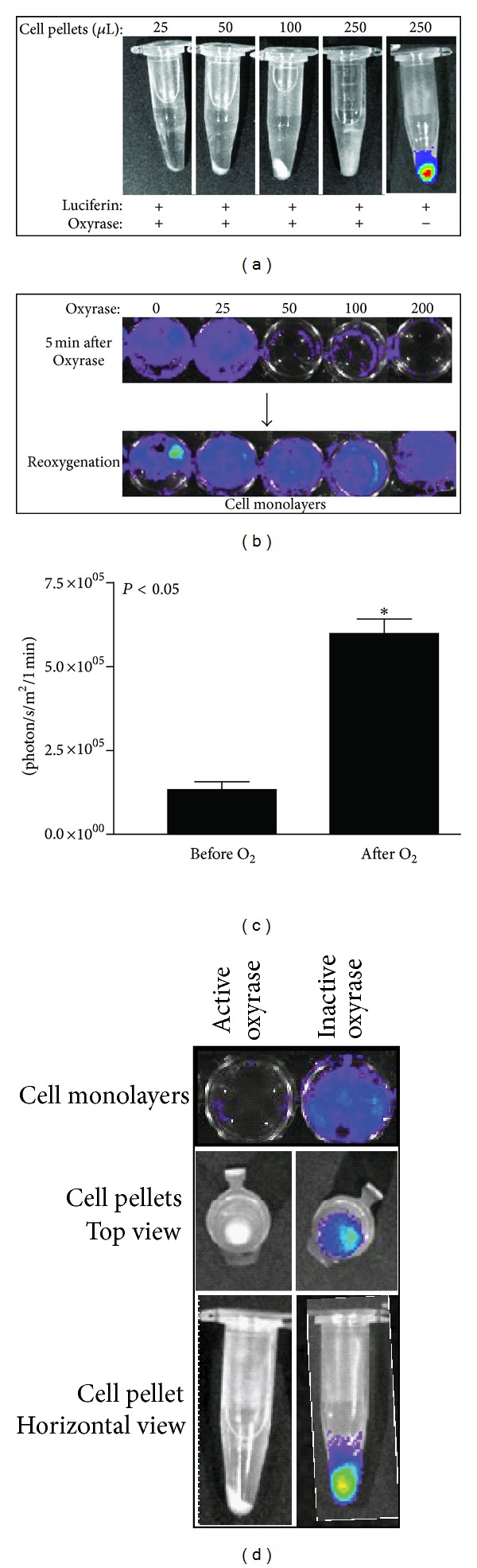
Effect of acute hypoxia and reoxygenation on BLS. U87-Luc cell pellets (a) or monolayers (b) were exposed to Oxyrase for 5 min to induce acute hypoxia before addition of luciferin and BLI. After Oxyrase addition and BLI, monolayers were reoxygenated by bubbling 1 mL of air through the media in each well; subsequent BLI is shown in (b) and results for the 200 mU/mL Oxyrase treatment are quantified in (c). Monolayers and cell pellets treated with Oxyrase that had been heat inactivated (boiling for 10 min) were assessed by BLI (d).

In order to investigate the effect of hypoxia on BLS from cultured cells, Oxyrase was used to create acute hypoxia. Oxyrase is a mixture of membrane monooxygenases and dioxygenases that removes dissolved oxygen rapidly from aqueous and semisolid environments [15, 16]. U87-Luc cell pellets and monolayers were exposed to Oxyrase for 5 min to induce acute hypoxia. Subsequent BLI is shown in Figure 3. Oxyrase caused a dose-dependent reduction in BLS with Oxyrase treatment. This reduction was reversed by reoxygenation (Figures 3(b) and 3(c)), which was performed by bubbling air through the media. Heat-inactivated Oxyrase did not impact BLS (Figure 3(d)).

**Figure 4:**
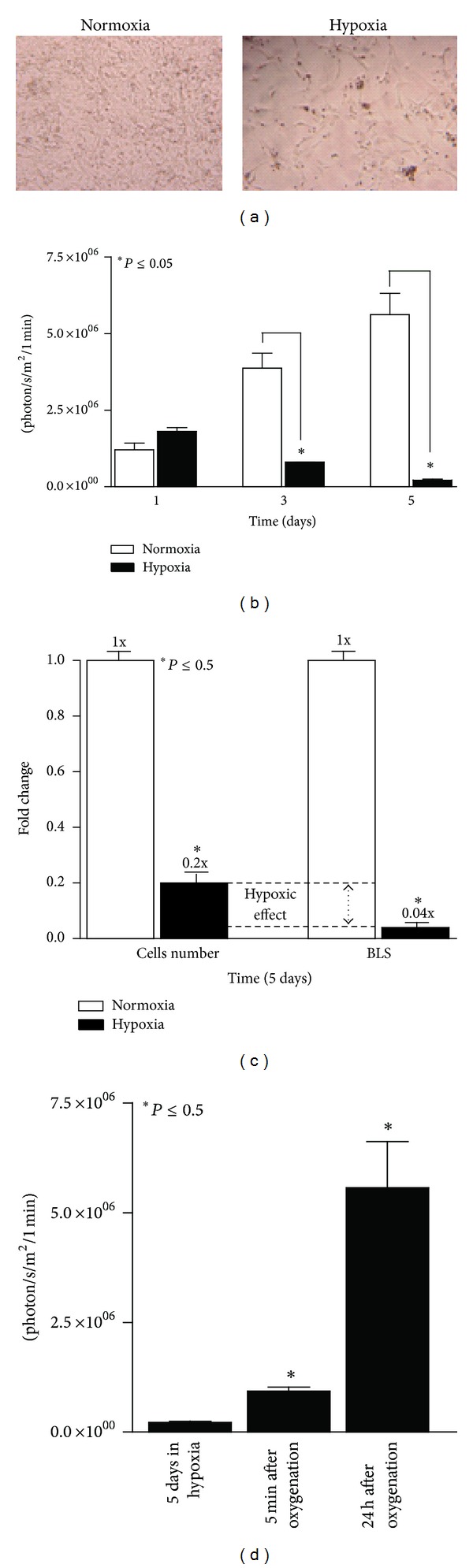
Effect of chronic hypoxia on BLS. U87-Luc cells were grown in 96-well plates either in normoxia or hypoxia (95% N_2_/5% CO_2_) for 5 d (phase contrast microscopy, (a)). BLI was performed on triplicate wells at 1, 3, and 5 d and quantified at 1 min (b). The impact of 5 d of hypoxia on cell number and BLS was compared quantitatively (c). For cells treated with hypoxia for 5 d, BLI was repeated 5 min and 24 h after re-oxygenation and quantified at 1 min (d).

To evaluate the effect of chronic hypoxia on BLI *in vitro*, U87-Luc cells were grown in either a normoxic or hypoxic environment for 5 days (Figure 4(a)) and BLI was performed at 1, 3, and 5 days. Cells grown in hypoxia showed statistically significant reduction in BLS compared to cells grown in normoxia (Figure 4(b)). While BLS was substantially reduced after 3 and 5 d in hypoxia, cell number also decreased; however, the reduction in BLS was much greater than the decrease in cell number, 25-fold versus 5-fold, respectively, suggesting a direct effect of hypoxia on the BLS (Figure 4(c)). At 5 min after reoxygenation, intensity of the BLS had quadrupled (*P* < 0.05) and it continued to dramatically increase for 24 h after reoxygenation (Figure 4(d)). The rapid early increase in BLS with re-oxygenation is not attributable to proliferation and represents the reversible impact of hypoxia on the luciferase reaction resulting in reduced light emission. Thus, the cell number is underestimated by the light emission under hypoxic conditions. At 24 h after re-oxygenation, further recovery of the BLS was noted and is likely attributable to both increased oxygen availability leading to “correction” of the BLS and also to proliferation in response to normoxia.

**Figure 5:**
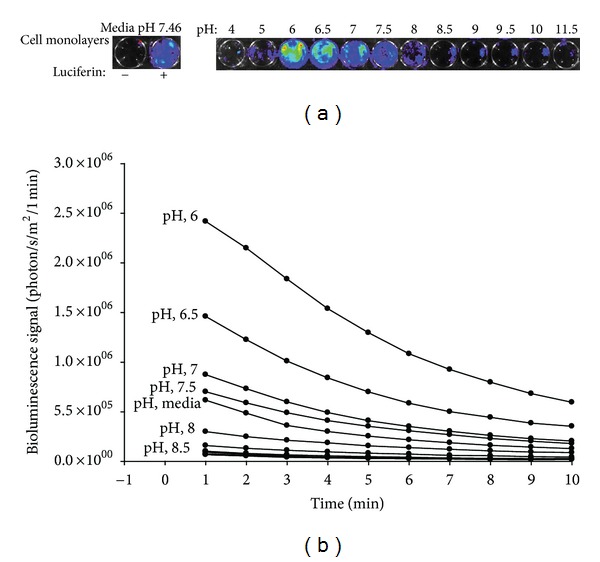
Influence of pH on BLS. U87-Luc monolayers were incubated in media with the indicated pH for one min. BLS images and quantification over time are shown in (a) and (b), respectively.

Hypoxia and acidosis may coexist in solid tumors, and many cellular processes are pH dependent, including enzymatic activity and cell proliferation [17]. In general, tumors are more acidic than normal tissues with median pH values of about 7.0 in tumors and 7.5 in normal tissues [18–20], and considerable variation in tissue pH at different regions of the same tumor has been observed [18].  Figure 5 demonstrates the impact of acidity on BLS from U87-Luc monolayers. BLS was maximal between pH 6 and pH 8, with a gradual decrease in BLS as the pH increased across this range.

**Figure 6:**
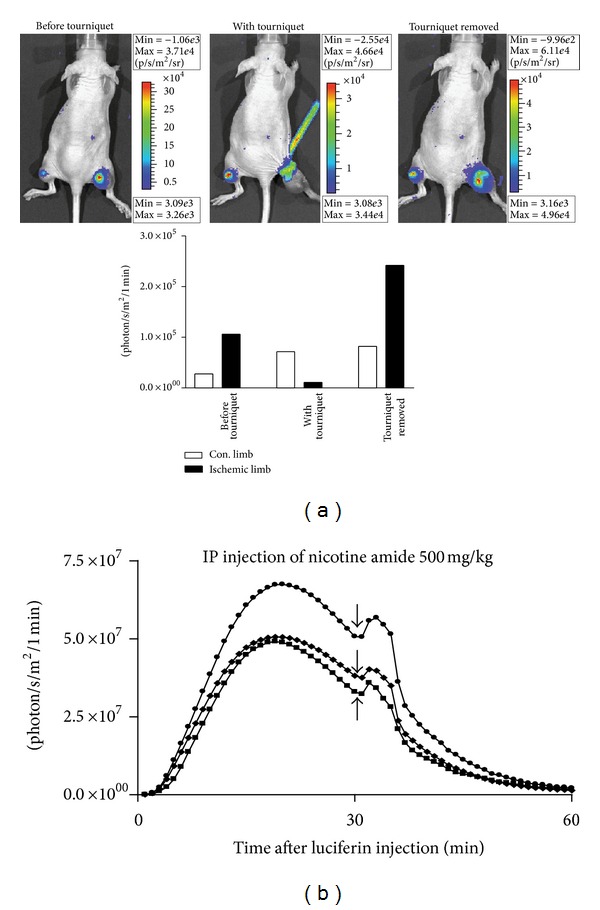
Effect of acute ischemia and reperfusion on *in vivo* BLS. (a) BLS was collected for 1 min in a nude mouse bearing bilateral U87-Luc subcutaneous thigh xenografts 5 min after injection of D-luciferin. The mouse was treated with a right thigh tourniquet proximal to the tumor. BLI was performed 2 min after tourniquet application and then 2 min after tourniquet removal. (b) BLS was collected at 1 min intervals for 60 min in nude mice bearing bilateral U87-Luc subcutaneous thigh xenografts. At 30 min, nicotinamide was administered via IP injection.

In order to demonstrate the effect of acute ischemia on *in vivo* BLI, a tourniquet was applied on the leg of a nude mouse proximal to an established U87-Luc thigh xenograft. BLI performed 2 min after placement of the tourniquet demonstrated substantially reduced BLS on the side of the tourniquet with stable BLS on the contralateral side. Within 2 min after releasing the tourniquet and restoring blood flow to the tumor, a strong BLS was present; the BLS was greater after reperfusion than prior to tourniquet placement (Figure 6(a)). Thus, acute ischemia results in underestimation of tumor size by BLI in an *in vivo* thigh xenograft tumor model. While the decreased blood flow following tourniquet placement undoubtedly leads to reduced substrate delivery to the tumor, adequate substrate was present to produce a strong BLS immediately prior to tourniquet placement (Figure 6(a)). Thus, the dramatic reduction in BLS following tourniquet placement is predominantly due to reduced oxygen tension in the tumor. This concept is supported by the finding that augmentation of tumor blood flow can enhance the BLS.  Figure 6(b) shows the BLS over time of U87-Luc thigh xenografts. At 30 min with declining BLS, the mice were given an intraperitoneal injection of nicotinamide, a peripheral vasodilator that increases tissue perfusion. Immediately after injection, BLS increased in all tumors, indicating that blood flow augmentation increases BLS.

**Figure 7:**
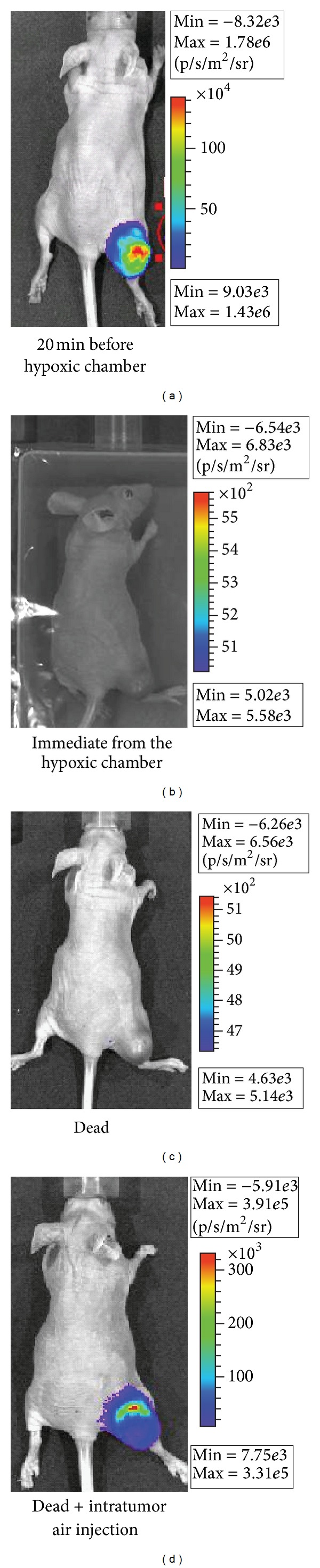
*In vivo* BLI after acute systemic hypoxia and cessation of blood flow. A nude mouse with a U87-Luc thigh xenograft tumor underwent BLI (a) before transfer to a hypoxic chamber (95% N_2_/5% CO_2_) for 10 min followed by BLI in a sealed bag (b). After euthanasia, BLI was performed (c). 1 mL of air was then injected into the tumor and BLI was repeated (d).

To further evaluate the significance of substrate delivery versus oxygenation, BLI was performed in a systemically hypoxic mouse, a dead mouse, and a dead mouse after intratumoral air injection. The results are shown in Figure 7. Prior to hypoxia, a strong BLS was noted (Figure 7(a)). After 10 min in the hypoxia chamber, the BLS was completely abolished (Figure 7(b)). When luciferin was injected prior to termination, the postmortem mice exhibited no BLS (Figure 7(c)). However, direct injection of air into the tumor resulted in a strong BLS similar to the premortem tumor. This demonstrates that, despite the lack of active perfusion and continuous substrate delivery that occurs in the post-mortem state, a BLS could still be generated by manual oxygenation. This suggests that a decrease in the BLS of hypoperfused tumors is not solely due to reduced substrate availability and that hypoxia plays an important role in reducing the BLS. Based on the previous data, tumors or treatments that involve hypoxia, hypoperfusion, or substantial pH changes may yield unreliable data when assessed by BLI. This may be especially important for tumors located in deep tissues [21]. It is assumed that the BLS will increase as the tumor volume increases, but this relationship may not hold in the setting of hypoxia. In certain cases, this may not lead to misinterpretation of treatment outcomes. For example, when solid tumors necrose centrally in response to therapy, the BLI signal will fall due to the hypoxia and necrosis, consistent with the “death” of a portion of the tumor despite the overall external tumor size continuing to enlarge as measured by calipers. In these cases, BLI may be more representative of treatment effect and caliper measurements may be misleading. Unfortunately, tumors that are simply hypoxic may also appear smaller by BLI, despite being adapted to grow at low oxygen tension. Tissue necrosis and death also affect the optical properties of tissues and thus affect scattering and absorption of light, which may additionally impact the interpretation of BLI.

## 4. Conclusions

The use of BLI in xenografts provides a convenient method for non-invasive monitoring of *in vivo *tumors; this is particularly useful when tumors are not accessible to calipers. Many solid tumors undergo a certain degree of hypoxia and pH changes with growth and/or treatment, which can significantly reduce the BLS. Thus, caution should be used in interpretation of BLI results when tumor hypoxia is present.
